# A Treasure for Families

**Published:** 1899-12

**Authors:** 


A TREASURE FOR FAMILIES. A Plea for Posterity and the Development of Marriage upon the Human Race. By Jas. W. Price, M.D

This little monograph is from the pen of Dr. Price, one of the oldest physicians in Atlanta. There is a great deal in it that is practical, and of great truth. In the present age too little is made of the sacredness of marriage, and this plea of Dr. Price's is timely in the extreme. We congratulate the doctor on his timely advice. The price of this little booklet is 25 cents, and is well worth the price.

